# Eco-Evolutionary Trophic Dynamics: Loss of Top Predators Drives Trophic Evolution and Ecology of Prey

**DOI:** 10.1371/journal.pone.0018879

**Published:** 2011-04-19

**Authors:** Eric P. Palkovacs, Ben A. Wasserman, Michael T. Kinnison

**Affiliations:** 1 Duke University Marine Laboratory, Beaufort, North Carolina, United States of America; 2 Nicholas School of the Environment, Duke University, Durham, North Carolina, United States of America; 3 School of Biology and Ecology, University of Maine, Orono, Maine, United States of America; Institute of Marine Research, Norway

## Abstract

Ecosystems are being altered on a global scale by the extirpation of top predators. The ecological effects of predator removal have been investigated widely; however, predator removal can also change natural selection acting on prey, resulting in contemporary evolution. Here we tested the role of predator removal on the contemporary evolution of trophic traits in prey. We utilized a historical introduction experiment where Trinidadian guppies (*Poecilia reticulata*) were relocated from a site with predatory fishes to a site lacking predators. To assess the trophic consequences of predator release, we linked individual morphology (cranial, jaw, and body) to foraging performance. Our results show that predator release caused an increase in guppy density and a “sharpening” of guppy trophic traits, which enhanced food consumption rates. Predator release appears to have shifted natural selection away from predator escape ability and towards resource acquisition ability. Related diet and mesocosm studies suggest that this shift enhances the impact of guppies on lower trophic levels in a fashion nuanced by the omnivorous feeding ecology of the species. We conclude that extirpation of top predators may commonly select for enhanced feeding performance in prey, with important cascading consequences for communities and ecosystems.

## Introduction

Top predator removal is reshaping communities and ecosystems at an alarming rate [1]. Nowhere are these changes more dramatic than in aquatic ecosystems, where predatory fishes are common targets of human harvest. Fisheries harvest has caused widespread and dramatic reductions in food chain length in both marine and freshwater ecosystems [2], [3], [4], [5], [6]. Top predator removal has received much attention for its ecological effects, especially in terms of trophic cascades, which have traditionally been linked to demographic and behavioral responses of prey populations [7], [8], [9], [10], [11]. It has also become widely recognized that fisheries harvest can cause direct evolutionary changes in harvested fish populations [12], [13]. However, the harvest of top predators, and the ensuing release from predation pressure, may also have important implications for prey evolution [14], [15] – and by extension the community and ecosystem properties affected by altered prey traits.

Recent theoretical and empirical research into eco-evolutionary dynamics suggests that contemporary evolution can have important effects on ecological processes [16], [17], [18]. In laboratory studies, evolution of prey populations in response to predators can cause major impacts on the ecological dynamics of aquatic systems [19], [20], [21]. One avenue for such effects is through an evolutionary trade-off between predation resistance and competitive ability. The effects of this trade-off have been explored in simple two-species rotifer-algae chemostat communities, where the reduction of rotifer predation pressure results in an overall increase in algal density that favors algal genotypes that are more efficient at acquiring resources [21], [22]. If such a response is common in nature, then the removal of top predators may not only drive increased prey density, but also cause enhanced prey resource use. Such a response in prey could serve to magnify the top-down effects of predator removal on lower trophic levels.

Here we examine the impact of predator release on the evolution of trophic morphology and feeding performance in wild populations of the Trinidadian guppy (*Poecilia reticulata* Peters 1859). The guppy is a model species for exploring the ecological and evolutionary implications of predator release in natural ecosystems [23], [24]. In habitats lacking fish predators, guppy density is generally higher and per capita resource availability lower than in habitats with predators [25], [26]. This scenario likely results in higher levels of intraspecific competition in low predation environments, which can impact the evolution of guppy life history traits [27]. Local adaptation in guppies to either the presence or absence of fish predators can also cause changes to stream communities and ecosystems [28], [29]. However, the impact of predator release and competition on the contemporary evolution of guppy trophic traits, and the potential consequences of these shifts for trophic interactions, are currently unknown.

To test whether release from predators has led to increased guppy population density and sharpened foraging traits, we took advantage of an introduction experiment initiated in 1976 where guppies were relocated from a site containing predatory fishes to a site lacking strong fish predators [30], [31]. We examined guppy density and foraging behavior and linked individual feeding performance to morphology. For behavior, we examined feeding performance in terms of the number of food items consumed during fixed-duration feeding trials. For morphology, we examined aspects of head shape (cranial and jaw morphology) and body shape (lateral profile) using landmark-based geometric morphometric analysis. These morphological traits influence both foraging efficiency and predator escape ability and are subject to performance trade-offs [32], [33], [34], [35]. By associating feeding performance with morphology at the individual level, we were able to identify the aspects of morphology that had the greatest impact on feeding mode and efficiency. We then tested whether those performance traits have diverged significantly over 32 years, or about 55 guppy generations, as a result of predator release. We predicted that predator release has increased guppy density and led to changes in trophic traits that have heightened top-down effects on the local food web. We assess these potential effects in light of related mesocosm and diet studies (i.e. [28], [29]).

## Materials and Methods

### Ethics Statement

The collection of fish was approved by the Ministry of Agriculture, Land and Marine Resources, Republic of Trinidad and Tobago. All handling of fish was approved by the University of Maine Institutional Animal Care and Use Committee (protocol A2005-06-08).

### Data Collection

In July 2008, guppies were sampled at three sites in the Aripo River drainage in the Northern Range Mountains of Trinidad: a natural high predation site (HP), a natural low predation site (LP), and a low predation site into which guppies were experimentally introduced in 1976 (GI). In terms of the coexisting fish community, all three sites contain *Rivulus hartii*, a guppy competitor and weak predator. Only the HP site contains the top fish predators in this system, *Crenicichla alta* and *Hoplias malabaricus.* The HP site was the source of the introduction, which involved about 200 guppies [30]. GPS locations and previous descriptions of sampling sites are provided in Appendix S1. Based on an estimate of 1.74 generations per year in LP environments [15], about 55 generations elapsed between the introduction and the collection of guppies for our study.

Guppy density was estimated using depletion sampling [36]. Within each site, the area designated for sampling was blocked at the upstream and downstream ends using seines, which prevented the movement of fish into or out of the sampling reaches during the sampling period. The length of each sampling reach was measured, as was the width of the reach at the upstream end, the downstream end, and the mid-point. These measurements were used to calculate the area sampled. A crew of 2-4 people netted guppies for 3-4 consecutive timed intervals, each lasting 15-40 min. At the end of each sampling interval, guppies were enumerated. Guppies were removed from the stream and held in buckets until all sampling intervals were complete. Sampling continued until either no guppies were captured during the final interval or until conditions precluded further sampling. In all cases, we were able to deplete the population of guppies in our sampling reaches such that catch-per-unit-effort depletion curves could be used to estimate population density. To do so, catch-per-unit-effort was plotted against the cumulative catch for each sampling interval and fitted with a linear relationship. The equation of this line was used to estimate initial population density.

We collected 20 adult female guppies from each site and transported them to the laboratory for behavioral assays. We examined only female guppies in this study because females, unlike males, have been found to lack phenotypic plasticity in trophic morphology (the same aspects of head and jaw shape examined here) resulting from alternate food presentations [34]. Therefore, any differences observed in trophic morphology are unlikely to be due to plasticity induced by guppy feeding mode. In contrast, variation in body shape for female guppies has been found to be influenced by local adaptation, phenotypic plasticity and pregnancy status [32], [37].

Guppies were held communally in 38 L tanks according to sampling locality until each was tested individually for feeding performance. Fish were fed standard flake food while in community tanks. Each fish was moved to a 9.5 L individual tank with no substrate at least 12 hours before it was tested. Fish underwent a fasting period of between 24 and 36 hours prior to testing to control for any prior effects of satiation. Each foraging trial was 10 min long. For all trials, the standard food source was previously frozen Chironimidae larvae. Chironimidae are a common aquatic food item for both HP and LP guppies in the wild [28]. Five larvae were placed haphazardly on the bottom of the tank (benthic food) and five were placed on the surface of the water (limnetic food). Both benthic and limnetic food items came from the same source, with the only difference being that limnetic items were dried at 250°C for approximately 30 minutes to reduce their water content and increase buoyancy. As such, the nutritional value of the alternate food presentations was equal.

We recorded the number of strikes (successful and unsuccessful) and whether strikes were directed toward limnetic items or benthic items. During the observation period we used the program JWatcher [38] as an events recorder. We used number of successful strikes (strikes leading to consumption) per 10 min trial as our metric of consumption rate. As an index of feeding specialization, we subtracted the number of successful strikes on benthic items from the number of successful strikes on limnetic items. A positive value represents specialization on limnetic food, and a negative value represents specialization on benthic food.

After feeding trials were concluded, fish were sacrificed in an overdose of tricaine methanesulphonate (MS-222) and fixed in 10% buffered formalin for approximately 2 months. Fish were then cleared and stained with Alizirin Red to highlight bony materials [39], [40]. Cleared and stained fish were photographed from two different positions. First, standard photographs of the left side of each fish were taken using a digital camera (D70, Nikon, Inc., New York, USA) over a light board with a ruler for scale. Second, dorsal photographs of the head of each specimen were taken at 8x magnification using a dissecting microscope (EZ4 D, Leica Microsystems, Inc., Illinois, USA). For all dorsal photographs, magnification was held constant, and a separate photograph of a ruler was used for scale.

**Figure 1:**
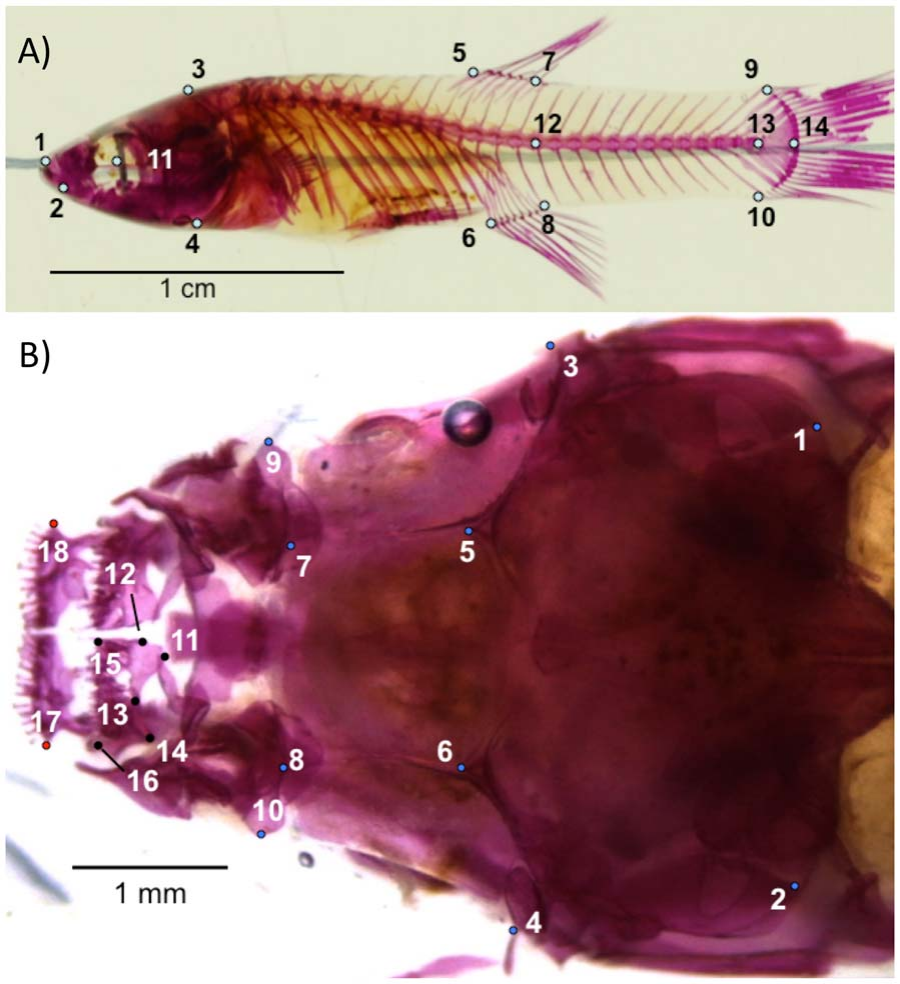
Landmarks for A) guppy body shape and B) guppy head shape. For the head, landmarks 1-10 represent cranial landmarks, landmarks 11-16 represent premaxillary landmarks, and landmarks 17-18 represent dentary landmarks. Full descriptions of landmark positions are provided in Appendix S2.

Landmarks for geometric morphometric analysis were digitized onto images using tpsDig2 version 2.14 [41]. Fourteen landmarks were digitized onto the lateral body photos of guppies (Fig. 1A, Appendix S2). A significant amount of variation in lateral body shape was due to bending of the fish during formalin fixation. We used tpsUtil version 1.44 [42] to “unbend” the specimens. This process involves fitting a quadratic curve along each fish's body through what should be a straight line, and then corrects for the arch in the entire data set. Landmarks 1 and 11-14 were used to define the quadratic curve. Further analyses were done on the adjusted coordinates for landmarks 1-10 only. Eighteen landmarks were digitized on the dorsal guppy head photographs (Fig. 1B, Appendix S2). All landmarks were treated as Type I (homologous), except for head landmark 13, which was treated as Type II (semi-sliding) in the analyses [43].

Articulated structures such as jaws present a problem to geometric morphometric techniques because movement of one body part relative to another introduces arbitrary variation into the calculation of shape variables. To account for this variation in our head shape dataset, we used the separate subsets method [44]. We split the head into three subsets: cranial (landmarks 1-10), premaxillary (landmarks 11-16), and dentary (landmarks 17 and 18). For the dentary, we used the inter-landmark distance to obtain a linear measure of length. To account for potential shape variation owing to relative size changes, we included the ratio of the centroid sizes of the premaxilla to the cranium. Since the photographs were taken perpendicular to the plane of articulation, we did not include the point of articulation in adjacent subsets in order to recreate thin plate spline deformations of the entire head data set. Instead, we display premaxillary and cranial deformations separately. Images are displayed next to each other, scaled approximately in order to aid in interpretation. We only digitized the left premaxillary bones; images are reflected across the medial axis of the head to aid in interpretation.

We used tpsRelw version 1.46 [45] to perform generalized least square Procrustes superimposition [46] to remove non-shape variation (the effects of rotation, translation, and the isometric effects of size). After superimposition, all further analyses were done separately on 20 body shape variables (superimposed x and y landmark coordinates) and 34 head shape variables (superimposed x and y cranial and premaxillary coordinates, dentary length, and the ratio of premaxillary to cranial centroid size). Since superimposition does not account for allometric effects of size, we included body centroid size as a covariate in our analyses [47], [48]. Our statistical results for shape variation were visualized using thin plate spline deformations in tpsSpline version 1.20 [49] using the consensus shape of the original fish as the reference.

### Statistical Analyses

Feeding rate (number of successful strikes per minute) and feeding specialization (number of successful strikes on limnetic food items minus number of successful strikes on benthic food items) were compared using a one-way analysis of variance (ANOVA) with site of origin as the factor. Tukey's Test for Honestly Significant Difference (HSD) was used to investigate *post hoc* pairwise contrasts. To determine if differences in feeding rate were due to differences in strike rate rather than feeding performance, we did two analyses. First we regressed feeding rate on strike rate (number of strikes taken per minute). Second we used a single-factor ANOVA to determine if strike rate differed among populations. All tests were performed separately at α = 0.05 and carried out in R version 2.10.1 unless otherwise noted.

To evaluate overall differences in morphology between populations, we performed a multivariate analysis of covariance (MANCOVA) for body and head data separately, with body centroid size as the covariate. We included an interaction term between body centroid size and population. We used jackknifed linear discriminant function analysis (DFA) to determine how well body and head shape discriminated among populations.

In order to test for the effect of morphology on an individual's performance, we used two-block partial least squares (2B-PLS) regression [50]. 2B-PLS is an extension of principal components analysis (PCA), which decomposes the data to find pairs of latent vectors that maximize the covariation between two sets (blocks) of data. We used 2B-PLS to identify the aspects of morphology that best predict feeding behavior. Our data blocks corresponded to a behavior block and a morphology block. We report 2B-PLS results separately for the body and head morphology, although a combined analysis showed qualitatively similar results. We determined the number of latent vectors to use by means of cross-validation [51]. Feeding performance and specialization were each used separately in the behavior block. 2B-PLS was performed using MATLAB. Significance was evaluated by means of permutation tests with 999 permutations plus the original data, making 1000 replicates [50]. We produced the deformation grids predicted by the extreme values of each latent vector. Further, we used analysis of covariance (ANCOVA), with body centroid size as the covariate, to test whether PLS scores – describing those aspects of morphology that best predicted feeding behavior – differed among populations. We included an interaction term between centroid size and population. Since centroid size was not significant in any of these tests, we removed the covariate and ran the model as a single factor ANOVA, with Tukey's HSD used for *post hoc* pairwise comparisons.

## Results

### Density, Behavior, and Overall Morphology

As predicted, guppy population density was highest at the LP site (20.9 guppies/m^2^), intermediate at the GI site (4.8 guppies/m^2^), and lowest at the HP site (0.4 guppies/m^2^). In feeding trials, guppies consumed an average of 0.29 food items per min (S.E. = 0.034), and feeding rates differed significantly among populations (ANOVA: F_2,45_ = 11.751, p<0.0001). LP and GI guppies consumed significantly more food items than HP guppies (Tukey's HSD: LP vs. HP, p<0.0001; GI vs. HP, p = 0.038), and LP guppies consumed marginally more food items than GI guppies (Tukey's HSD: LP vs GI, p = 0.077). Strike rate was not a significant predictor of feeding rate (Linear Regression: F_1,43_ = 0.600, p = 0.441) and did not differ between populations (ANOVA: F_2,43_ = 1.160, p = 0.324). Overall, guppies showed a slight but non-significant preference for limnetic food (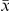  = 0.5625, S.E. = 0.3484; t = 0.538, p = 0.593). However, specialization did not differ among populations (ANOVA: F_2,45_ = 1.276, p = 0.289). Overall body and head shape differed significantly among populations (Table 1). Interactions between centroid size and population were not significant. DFA based on body and head shape correctly assigned 54.2% and 41.2% of guppies to one of the three population sources, respectively.

**Table 1:** Results of MANCOVA analyses for guppy head and body shape.

	Wilks' λ	*F*	*df* (numerator, denominator)	*P*	% Partial Variance Explained
Guppy Body					
Population	0.0809	2.8924	40,46	0.0003	0.7156
Body Centroid Size	0.1888	4.9427	20,23	0.0002	
Interaction	0.2263	1.2673	40,46	0.2181	
Guppy Head					
Population	0.0088	2.5574	68,18	0.0144	0.9062
Body Centroid Size	0.0124	21.0000	34,09	<0.0001	
Interaction	0.0526	0.8897	68,18	0.6502	

### Functional Morphology

**Figure 2:**
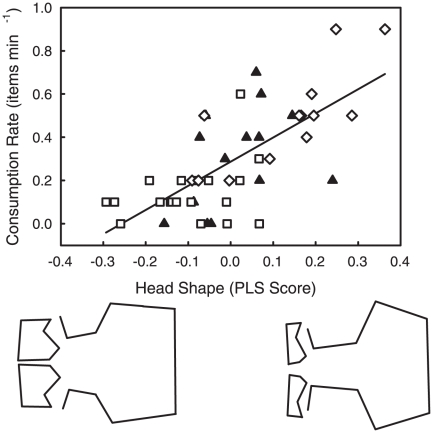
Head shape (PLS score) plotted against food consumption rate for high predation (open squares), low predation (open diamonds), and introduced (filled triangles) guppy populations. Head shape was a significant predictor of feeding rate. All three populations were significantly differentiated along the “feeding performance” axis. In the introduced population, the morphological features that enhanced foraging performance evolved away from the original high predation state and towards the natural low predation state. Deformation images represent the extremes of the PLS vectors exaggerated by a factor of 3 to aid in interpretation.

**Figure 3:**
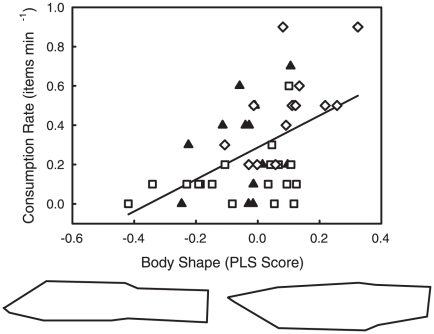
Body shape (PLS score) plotted against food consumption rate for high predation (open squares), low predation (open diamonds), and introduced (filled triangles) guppy populations. Body shape was not a significant predictor of feeding rate. The low and high predation populations were significantly different, while the introduced population did not differ from either. Deformation images represent the extremes of the PLS vectors exaggerated by a factor of 3 to aid in interpretation.

Guppy head shape was a significant predictor of feeding rate (Fig. 2; 2B-PLS: R^2^ = 0.49, p = 0.001). Guppies with high consumption rates had wider mouths, smaller and thinner premaxillae, shorter and wider crania, and larger, more dorsal eyes (Fig. 2). In contrast, guppies with low consumption rates had narrower mouths, thicker premaxillae, longer crania, and smaller, less dorsal eyes. PLS scores for feeding rate on head shape differed significantly among populations (ANOVA: F_2,43_ = 13.862, p<0.0001). All three populations were significantly differentiated along the “feeding performance” axis such that LP > GI > HP (Fig. 2; Tukey's HSD: HP vs. LP, p<0.0001; HP vs. GI, p = 0.029; GI vs. LP, p = 0.046). Guppy body shape was not a significant predictor of feeding rate (Fig 3; 2B-PLS: R^2^ = 0.25, p = 0.106). However, guppies with higher food acquisition rates tended to have deeper bodies, a more dorsal mouth position, and shorter caudal peduncles that tapered posteriorly. In contrast, guppies with low consumption rates had shallower bodies, more downturned mouths, and longer, less tapered caudal peduncles. PLS scores for feeding rate on body shape differed significantly among populations (ANOVA: F_2,43_ = 4.352, p = 0.019). The LP population had a greater mean PLS score than the HP population, while the GI population did not differ from either (Fig. 3; Tukey's HSD: HP vs. LP, p = 0.022; HP vs. GI, p = 0.973; GI vs. LP, p = 0.055). Neither head shape nor body shape was a significant predictor of trophic specialization on limnetic vs. benthic food items (head shape PLS: R^2^ = 0.2565, p = 0.329; body shape PLS: R^2^ = 0.1569, p = 0.515). Because specialization did not differ significantly among populations and because morphology was not a significant predictor of specialization, we did not test whether PLS scores based on trophic specialization differed among populations.

## Discussion

Fisheries harvest is extirpating top predators from marine and freshwater ecosystems on a global scale [1], [2], [3], [4], [5], [6]. While much effort has gone into understanding the effects of fisheries harvest on trophic interactions in truncated food chains [9], [10], [11] and on the contemporary evolution of harvested populations themselves [12], [13], almost nothing is known about the indirect effects of predator removal on prey evolution and its consequences for trophic interactions. Predator release may heighten intraspecific competition in prey by increasing prey density and decreasing per capita resource availability [52], [53], [54]. Laboratory studies of eco-evolutionary dynamics have shown that, when prey species face an evolutionary trade-off between predator avoidance and competitive ability, release from predation can sharpen the resource use traits of prey, with implications for ecological interactions [21], [22], [55], [56]. However, such effects have not previously been explored in the wild.

Here we examined the effect of predator release on the contemporary evolution of morphology and feeding performance in an experimentally introduced guppy population. We utilized an analysis technique that directly links individual variation in morphology to feeding performance and tested whether functional divergence in morphology and food acquisition rate has occurred since the introduction, as a result of predator release. Our results show that guppy behavior and morphology diverged significantly over 32 years or about 55 generations. In the introduced population, feeding performance and morphology shifted away from the original HP state and towards to natural LP state. As predicted, contemporary changes in morphology coincided with an increase in guppy population density and were linked to heightened food acquisition rates.

The morphological features that enhanced guppy foraging performance included a wider gape, a thinner premaxilla, a shorter, wider cranium and larger, more dorsal eyes (Fig. 2). Wider gapes and thinner premaxillae may enhance feeding performance by increasing the target size and jaw speed, respectively. Wider crania may be necessary to accommodate wider gapes. Larger, more dorsal eyes may be more acute for detecting potential food items; however, a more dorsal eye position was neither associated with enhanced performance on limnetic food nor decreased performance on benthic food, as might be expected. Overall guppy body shape also changed as a result of predator release (Table 1), but body shape was not a significant predictor of feeding behavior (Fig 3).

In Poeciliid fishes, evolutionary trade-offs between predator avoidance and resource acquisition may be found in aspects of head and body shape [32], [33]. O'Steen et al. [57] examined the contemporary evolution of predator escape ability in several guppy introduction experiments, including the set of populations we utilized here. They reared guppies in common gardens and found that introduced guppy populations rapidly lost their escape ability when introduced into low predation habitats. They attributed these declines to fitness trade-offs. Our results support this conclusion and point to a specific trade-off between escape ability and foraging ability. We found that a more dorsal eye position and larger head are better for the rapid consumption of food items (Fig 2), and that guppies with the highest consumption rates tended to display deeper bodies and smaller caudal peduncles, although the effect of body shape on food acquisition rate was not statistically significant (Fig 3). However, the opposite traits – a more ventral eye position, smaller head, thinner body, and larger caudal peduncle – are advantageous for escaping predators [35], [58], [59]. Thus, when predators are present and resources abundant, natural selection for gathering resources may be superseded by selection for avoiding predation, at a cost to foraging performance. In this way, natural selection driven by predators may indirectly limit foraging efficiency and alter cascading trophic interactions by favoring traits that enhance escape performance. Overall, release from predation appears to increase population density, intensify intraspecific competition, and thereby favor traits that facilitate the acquisition of scarce food resources.

We utilized wild-caught female guppies in this experiment, not individuals born in a common garden, and therefore cannot exclude the possibility of phenotypic plasticity influencing our results. However, current evidence does not support plasticity as a major factor underlying variation in trophic morphology for female guppies. Indeed, Robinson and Wilson [34] failed to induce plasticity in female guppies with different food presentations (different orientations and positions in the water column). Their study utilized juvenile guppies from a low-predation population, born in a common environment, and exposed to different feeding treatments beginning at less than one week of age. Thus, they provided a fairly stringent test of plasticity, albeit under a specific set of conditions. Plasticity in trophic morphology induced by other environmental variables, such as predator cues, and among-population variation in the degree of plasticity are additional factors that require evaluation.

In terms of body shape, we cannot directly account for difference in stream flows, which can cause plasticity [60], [61]. Nor can we exclude the possible effects of correlated selection on other traits, most notably life history traits. There is evidence that flow differences can cause phenotypic divergence in body shape in guppies [37], but the degree to which those differences are genetic versus plastic has not been directly tested. In terms of correlated selection, it has been noted that life history divergence may contribute to population differences in guppy body shape [32], [37]. Thus, some aspects of body shape divergence noted here could be the byproduct of local adaptation in reproductive investment. However, reproductive allocation itself is also molded by tradeoffs between selection for reproduction and selection for mobility required in foraging and escaping predators [62], [63].

While it is important to understand the myriad of interacting factors underlying phenotypic differences in wild populations, it is the phenotypes themselves that interact in food webs. Therefore, regardless of the causes of phenotypic divergence noted in our study, the consequences for trophic interactions are likely to be important. We found that release from predation is associated with contemporary sharpening of trophic traits. But how general is this result? At a broad scale, our findings are consistent with the algal-rotifer laboratory studies, where competitive phenotypes are associated with reduced predation pressure and high conspecific abundance [21], [22]. For guppies, our findings are expected to be general to the extent that a common tradeoff exists between escape ability and foraging ability. As noted, O'Steen et al. [57] have previously shown that predator release drove the rapid loss of guppy escape ability in multiple introduction experiments (including the one examined here). Therefore, we would predict concomitant increases in foraging performance in each of these introductions.

The sharpening of guppy trophic traits might be expected to intensify the top-down effects of guppies on stream ecosystems. The specific form of such effects, however, must be considered in light of the omnivorous trophic ecology of this species. Results from prior mesocosm studies have suggested that LP guppies do not cause more dramatic top-down effects on invertebrates compared to HP guppies; however, LP guppies do consistently reduce algal standing stocks compared to HP guppies [28], [29]. Therefore, the higher feeding rates of LP guppies may be primarily directed towards a greater consumption of algal resources. This notion is supported by analysis of diets in both mesocosms and in the wild [28], [29]. Interestingly, top-down trophic traits may not be the only mechanism by which prey evolution leads to ecological effects at other trophic levels. In guppies, predator release drives contemporary evolution of life history traits, increasing age and size at maturity [14], [15]. Excretion studies show that increases in the age and size structure of a population reduce nutrient recycling rates, which may then reduce algal production through bottom-up effects [29]. Thus, predator removal may decrease primary production by increasing guppy density (a top-down ecological effect), increasing consumption (a top-down evolutionary effect), and decreasing nutrient excretion (a bottom-up evolutionary effect). In this way, evolution may serve to “amplify” the strength of trophic cascades.

As human activity continues to decimate top predator populations worldwide, it will be increasingly important to understand potential implications for food webs [1]. While numerous direct and indirect ecological effects are expected, our results suggest that evolutionary effects should not be overlooked. Ample examples already demonstrate that harvest can result in the evolution of harvested species [12], [13]. Here we show that predator removal may also drive the evolution of functional traits in prey. Such cascading evolutionary effects of predator removal have the potential to alter ecological interactions and impact trophic dynamics. Predicting the ecosystem implications of top predator removal may therefore require a detailed understanding of contemporary evolution in prey, and perhaps knowledge of evolution across the diversity of species linked in food webs.

## Supporting Information

Appendix S1 **Sampling locations in the Aripo River drainage of Trinidad.**
(PDF)

Appendix S2 **Descriptions of landmarks for geometric morphometric analyses.**
(PDF)
