# Supplementary material for: Eco-Evolutionary Trophic Dynamics: Loss of Top Predators Drives Trophic Evolution and Ecology of Prey
Source: PLoS One. 2011 Apr 19;6(4):e18879. doi: 10.1371/journal.pone.0018879 (PMC3079755; doi:10.1371/journal.pone.0018879)
Supplement: Appendix S1 — Sampling locations in the Aripo River drainage of Trinidad. (PDF) [file pone.0018879.s001.pdf]

Appendix S1. Sampling locations in the Aripo River drainage of Trinidad. GPS coordinates and previous site names (from [30,31]) are given.

Site	Latitude	Longitude	Previous Description
HP	10.66568N	61.22789W	Aripo 6 (source of introduction)
LP	10.69048N	61.23689W	Aripo 1
GI	10.67111N	61.23203W	Aripo I
