# Supplementary material for: Eco-Evolutionary Trophic Dynamics: Loss of Top Predators Drives Trophic Evolution and Ecology of Prey
Source: PLoS One. 2011 Apr 19;6(4):e18879. doi: 10.1371/journal.pone.0018879 (PMC3079755; doi:10.1371/journal.pone.0018879)
Supplement: Appendix S2 — Descriptions of landmarks for geometric morphometric analyses. (PDF) [file pone.0018879.s002.pdf]

Appendix S2. Descriptions of landmarks for geometric morphometric analyses.

A) Body landmarks: (1) tip of the snout, (2) intersection of the premaxilla and the anguloarticular bones, (3) dorsal posterior edge of the cranium, (4) inflection point where operculum intersects the fish outline, (5) anterior extent of the dorsal fin insertion, (6) anterior extent of anal fin insertion, (7) posterior extent of the dorsal fin insertion, (8) posterior extent of the anal fin insertion, (9) dorsal extent of the caudal fin insertion, (10) ventral extent of the caudal fin insertion, (11) center of the orbit, (12) 13th vertebra from the posterior end, (13) most posterior vertebra, and (14) posterior tip of the hypural plate.

B) Head landmarks: Cranium: (1, 2) posterior cranium, (3, 4) posterior infraorbitals, (5, 6) posterior corners of the cranial plate, (7, 8) anterior corners of the cranial plate, (9, 10) anterior infraorbitals. Premaxilla: (11) posterior extent of the ascending process, (12) upwards projection on the medial edge of ascending process, (13) point on the posterior edge of the premaxilla at its narrowest point, (14, 15, 16) posterodistal, anteromedial, and anterodistal extents of the descending process, respectively. Dentary: (17, 18) ends of the dentary bone (used to calculate interlandmark distance).
